# Trends in List Prices, Net Prices, and Discounts for Originator Biologics Facing Biosimilar Competition

**DOI:** 10.1001/jamanetworkopen.2019.17379

**Published:** 2019-12-13

**Authors:** Alvaro San-Juan-Rodriguez, Walid F. Gellad, Chester B. Good, Inmaculada Hernandez

**Affiliations:** 1 Department of Pharmacy and Therapeutics, University of Pittsburgh School of Pharmacy, Pittsburgh, Pennsylvania; 2 Division of General Internal Medicine, University of Pittsburgh School of Medicine, Pittsburgh, Pennsylvania; 3 Center for High-Value Health Care, Insurance Services Division, UPMC Health Plan, Pittsburgh, Pennsylvania

## Abstract

In this cohort study, pricing data from January 2007 to June 2018 from SSR Health were used to determine how list prices, net prices, and discounts for the originator biologics changed with biosimilar competition.

## Introduction

Biosimilars hold promise for reducing spending on biologics.^1^ Biosimilar prices are estimated to be 15% to 16% lower than originator products,^2^ but it is unclear how list prices, net prices, and discounts for the originator products change with biosimilar competition.

We selected filgrastim, pegfilgrastim, infliximab, and insulin glargine as case studies because these were the only originator biologics that, by December 2018, faced competition from biosimilars (filgrastim-sndz, filgrastim-aafi, pegfilgrastim-jmdb, infliximab-dyyb, and infliximab-abda) or from other within-molecule substitutes (tbo-filgrastim and insulin glargine [Basaglar; Lilly]).^3^

## Methods

We obtained pricing data from January 2007 to June 2018 from SSR Health,^4^ which provides quarterly estimates of list prices, net prices, Medicaid discounts, and discounts in other payers for branded products with US sales reported by publicly traded companies. Quarterly net prices are calculated as the ratio between sales and number of units sold. Discounts are calculated as the ratio between the difference in list and net prices (numerator) and the list prices (denominator). Net prices and discounts capture all manufacturer concessions and not solely rebates.^4^ For each drug and year, we calculated the mean list and net prices and the mean discounts in Medicaid and other payers.^5^

We conducted our analyses from August 12 to October 7, 2019. This study was approved by the institutional review board at the University of Pittsburgh as exempt from informed consent procedures because of the use of unidentifiable data. We followed the Strengthening the Reporting of Observational Studies in Epidemiology (STROBE) reporting guideline for cohort studies.

## Results

**Figure 1.**
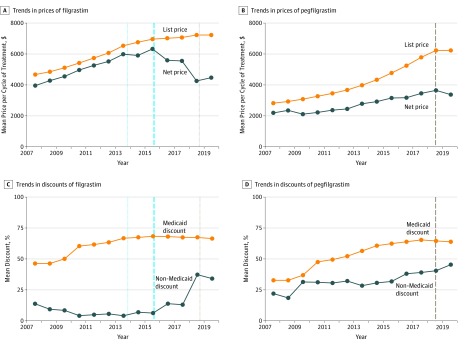
Trends in List Prices, Net Prices, and Discounts of Originator Filgrastim and Pegfilgrastim, 2007-2019 A and B, The 2007 to 2019 trends in list and net prices of originator filgrastim (A) and pegfilgrastim (B) are shown. We defined price of treatment, based on the dosing recommendations of the US Food and Drug Administration, as the mean price of treating a standard 80-kg patient per cycle of treatment. C and D, Mean discounts in Medicaid and in payers other than Medicaid from 2007 to 2019 for filgrastim (C) and pegfilgrastim (D) are shown. The blue dashed line (filgrastim-sndz) and the brown dotted line (filgrastim-aafi) in panels A and C, as well as the brown dashed line (pegfilgrastim-jmdb) in panels B and D, represent the entry of biosimilar competitors, whereas the blue dotted line (tbo-filgrastim) in panels A and C represents the entry of a competitor approved through a pathway other than the abbreviated biosimilar licensure pathway. Tbo-filgrastim was not approved through the biosimilar pathway; however, it constitutes a substitute for originator filgrastim because it presents the same molecule and formulation. Estimates of net prices and discounts comprise all concessions made by manufacturers, including rebates, coupon cards, 340B discounts, prompt pay discounts, returns provisions, and any other deductions accounted for in the reporting of net sales.^4^ SSR Health estimated Medicaid discounts as the sum of the 23.1% rebate and the inflation rebate for price increases above the consumer price index.^5^ Discounts in payers other than Medicaid were estimated by subtracting Medicaid discounts from total discounts and the number of units reimbursed by Medicaid from total US sales. Thus, supplemental Medicaid rebates negotiated by states or managed care organizations are captured by estimates of discounts in payers other than Medicaid rather than by estimates of Medicaid discounts.

List and net prices of filgrastim increased in parallel each year by a mean of 5.1% (95% CI, 4.0%-6.2%) and 6.1% (95% CI, 3.7%-8.4%), respectively, until the introduction of its first biosimilar in 2015, when list prices stagnated and net prices commenced to decrease by a mean of −7.7% (95% CI, −19.9% to 4.6%) annually (Figure 1). Similarly, list and net prices of pegfilgrastim increased annually by a mean of 7.5% (95% CI, 6.2%-8.8%) and 4.9% (95% CI, 1.3%-8.5%), respectively, until the introduction of its first biosimilar in 2018, when list prices stagnated and net prices decreased by −7.4% (−$269.2 of $3640.1).

**Figure 2.**
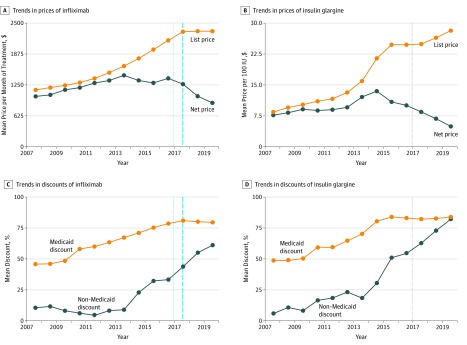
Trends in List Price, Net Price, and Discounts of Originator Infliximab and Insulin Glargine, 2007-2019 A and B, The 2007 to 2019 trends in list and net price of originator infliximab (A) and insulin glargine (B) are shown. We defined cost of treatment, based on the dosing recommendations of the US Food and Drug Administration, as the mean price of treating a standard 80-kg patient per month of treatment for infliximab and as the mean cost per 100 international units (IU) for insulin glargine. C and D, Mean discounts in Medicaid and in other than Medicaid from 2007 to 2019 for infliximab (C) and insulin glargine (D). The vertical lines in panels A and C represent the entry of biosimilar competitors (blue dotted line, infliximab-dyyb; blue dashed line, infliximab-abda), and the brown dotted line in panels B and D represents the entry of a competitor approved through a pathway other than the abbreviated biosimilar licensure pathway—insulin glargine (Basaglar [Lilly]). Insulin glargine (Basaglar) was not approved through the biosimilar pathway; however, it constitutes a substitute for originator insulin glargine because it presents the same molecule and formulation. Estimates of net prices and discounts comprise all concessions made by manufacturers, including rebates, coupon cards, 340B discounts, prompt pay discounts, returns provisions, and any other deductions accounted for in the reporting of net sales.^4^ SSR Health estimated Medicaid discounts as the sum of the 23.1% rebate and the inflation rebate for price increases above the consumer price index.^5^ Discounts in payers other than Medicaid were estimated by subtracting Medicaid discounts from total discounts and the number of units reimbursed by Medicaid from total US sales. Thus, supplemental Medicaid rebates negotiated by states or managed care organizations are captured by estimates of discounts in payers other than Medicaid rather than by estimates of Medicaid discounts.

List and net prices of infliximab increased in parallel by a mean of 6.0% (95% CI, 4.2%-7.8%) and 6.0% (95% CI, 3.9%-8.2%), respectively, from 2007 to 2013 (Figure 2). In 2013, net prices started to decrease by a mean of −1.3% (95% CI, −9.7% to 7.2%) per year, and decreases further accelerated to a mean of −13.6% (95% CI, −19.7% to −7.5%) per year after the introduction of its first biosimilar in 2017. From 2007 to 2014, list and net prices of insulin glargine increased annually by a mean of 14.7% (95% CI, 7.1%-22.3%) and 8.8% (95% CI, 1.9%-15.6%), respectively. From 2015 to 2019, list price growth slowed to a mean of 5.8% (95% CI, 0.4%-11.2%) annually. Net prices started to decrease by a mean of −14.4% (95% CI, −21.1% to −7.7%) annually in 2015 but accelerated to a mean of −23.5% (95% CI, −31.2% to −15.7%) after the entry of a substitute in 2017.

Across the study period, Medicaid discounts increased by 20.1 (95% CI, 19.4-20.7) percentage points for filgrastim, by 31.2 (95% CI, 30.9-31.5) percentage points for pegfilgrastim, by 33.8 (95% CI, 33.1-34.4) percentage points for infliximab, and by 35.2 (95% CI, 34.2-36.2) percentage points for insulin glargine. In payers other than Medicaid, discounts increased by 20.3 (95% CI, 12.1-28.5) percentage points for filgrastim, by 23.4 (95% CI, 15.6-31.2) percentage points for pegfilgrastim, by 50.6 (95% CI, 49.4-51.7) percentage points for infliximab, and by 76.1 (95% CI, 74.1-78.1) percentage points for insulin glargine.

## Discussion

In 4 case studies, we observed that the net prices of originator biologics decreased following the entry of biosimilars or other substitutes. For infliximab and insulin glargine, decreases in net prices had commenced 2 to 3 years before the commercialization of competitors; however, decreases further accelerated following competitor entry. Decreases in net prices were primarily due to increases in manufacturer discounts, specifically in payers other than Medicaid.

While the decreasing net prices of infliximab and filgrastim had been previously described,^6^ our study is the first, to our knowledge, to examine pegfilgrastim and insulin glargine and the contribution of non-Medicaid discounts toward lowering net prices.

Our study is subject to several limitations. First, estimates of Medicaid discounts only reflect the statutory rebates. Second, our analyses cannot determine whether net price decreases were associated with the entry of biosimilars or with other factors. Third, there are important differences in the market of each of the agents included in our study; for instance, insulin glargine is not considered a specialty drug, and infliximab is mostly administered in physician’s offices. Fourth, it is not possible to establish whether larger discounts were due to increases in rebates or to other manufacturer concessions. Nevertheless, our findings show that biologics that faced biosimilar competition—even without interchangeability—showed marked reductions in net prices and leveling off of list price increases.
